# Assessment of quality of life in HAART-treated HIV-positive subjects with body fat redistribution in Rwanda

**DOI:** 10.1186/1742-6405-4-19

**Published:** 2007-09-18

**Authors:** Eugene Mutimura, Aimee Stewart, Nigel J Crowther

**Affiliations:** 1 Faculty of Allied Health Sciences & Programs in HIV/AIDS Clinical Research and Community Interventions, Kigali Health Institute, B. P 3286 Kigali, Rwanda; 2 School of Therapeutic Sciences, University of the Witwatersrand, Johannesburg, Republic of South Africa; 3 Department of Chemical Pathology, National Health Laboratory Service, University of the Witwatersrand, Johannesburg, Republic of South Africa

## Abstract

**Background:**

The introduction of HAART has initially improved the quality of life (QoL) of HIV-positive (HIV+) patients, however body fat redistribution (BFR) and metabolic disorders associated with long-term HAART use may attenuate this improvement. As access to treatment improves in sub-Saharan Africa, the disfiguring nature of BFR (peripheral atrophy and/or central adiposity) may deter treatment adherence and initiatives and decrease QoL. We examined the relationship between BFR and domains of QoL in HAART-treated HIV+ African men and women with (HIV+BFR, n = 50) and without (HIV+noBFR, n = 50) BFR in Rwanda.

**Results:**

HIV+ subjects with BFR were less satisfied with their body image (4.3 ± 0.1 versus 1.5 ± 0.2; p < .001), self-esteem and social life (4.1 ± 1.4 versus 2.1 ± 0.3; p = 0.003). HIV+BFR were more ashamed in public (4.5 ± 1.2 versus 1.1 ± 1.1), reported less confident about their health (4.6 ± 1.4 versus 1.5 ± 1.2) and were frequently embarrassed due to body changes (4.1 ± 1.1 versus 1.1 ± 0.9) (p < .001) than HIV+noBFR. HIV+ Rwandan women with BFR reported more dissatisfaction with psychological (8.3 ± 2.9 versus 13.7 ± 1.9), social relationships (6.9 ± 2.3 versus 11.1 ± 4.1) and HIV HAART-specific domain of wellbeing (3.1 ± 4.8 versus 6.3 ± 3.6) (p < .001). Age was associated with independence (r^2 ^= 0.691; *p *= 0.009) and marital status was associated with psychological (r^2 ^= 0.593; *p *= 0.019) and social relationships (r^2 ^= 0.493; *p *= 0.007). CD4 count (r^2 ^= 0.648; *p *= 0.003) and treatment duration (r^2 ^= 0.453; *p *= 0.003) were associated with HIV HAART-specific domain of wellbeing. HIV+ Rwandan women with BFR were significantly more affected by abdominal adiposity (p < .001), facial and buttocks atrophy (p < .05) than HIV+ men with BFR.

**Conclusion:**

Body fat alterations negatively affect psychological and social domains of quality of life. These symptoms may result in stigmatization and marginalization mainly in HAART-treated African women, adversely affecting HAART adherence and treatment initiatives. Efforts to evaluate self-perceived body fat changes may improve patients' wellbeing, HAART adherence and treatment outcomes and contribute towards stability in quality of life continuum.

## Background

The psychological and social effects of Human Immunodeficiency Virus (HIV) and Acquired Immune Deficiency Syndrome (AIDS) on patients' quality of life (QoL) have been constantly fluctuating [1]. With the advent of highly active antiretroviral therapy (HAART), people affected with HIV/AIDS can now live a longer thriving life [2]. The World Health Organization (WHO) '3 by 5', the Presidential Emergency Program for AIDS Relief (PEPFAR), bilateral and multilateral agencies have facilitated resource-limited countries including sub Saharan Africa to scale-up HIV treatment, maintaining effective and appropriate standard of care [3]. As a result of WHO guidelines and global initiatives, HIV treatment efficacy in resource-limited countries is promising [4]. Although the benefits of HIV treatment are well established [2], the use of HAART in approximately 40–60% of patients, has been linked to a constellation of treatment challenges, including metabolic abnormalities and body fat redistribution (BFR), often called HIV lipodystrophy [5,6].

The characteristics of BFR resulting in distinct abnormal fat gain for the neck (buffalo hump), abdomen, breasts, and/or fat loss for the face, limbs and buttocks are considerably disfiguring [7]. BFR compromises HIV serostatus privacy, producing social isolation and distress, resulting in psychological repercussions [8]. In fact, HIV patients with BFR would trade off length of life or accepted greater risks of mortality in order to maintain a life free of body fat alterations [9]. Therefore, BFR may influence patients' beliefs about benefits of HAART, and decrease adherence with subsequent adverse impact on patients' QoL [10]. Studies on QoL and BFR in HIV+ sub Saharan patients are limited. Information regarding the relationship between BFR and QoL is important as access to HAART for HIV+ patients in sub Saharan countries is steadily improving. Therefore, we examined the relationship between BFR and QoL in HAART-treated HIV+ African men and women with BFR in Rwanda.

## Methods

### Subjects

Subjects with HIV infection on stable HAART were enrolled in the study from August 2005 to July 2006. Subjects attended routine medical follow-up and periodic CD4 cell counts from tertiary health centres of Centre Hospitalier Universitaire de Kigali, Treatment & Research AIDS Centre and Centre Hospitalier Universitaire de Butare. Subjects were also recruited from HIV/AIDS clinics of Kimironko, Kicukiro, Bilyogo-Nyiranuma, Kinyinya and Kacyiru Health Centres. Information regarding the type and duration of HAART was obtained from subjects' dispensation cards. Eligible subjects had documented HIV infection, were 21–50 years and on stable WHO-recommended HAART ≥ 6 months. Subjects had no active opportunistic infections or significant symptoms of HIV disease which could adversely influence their expectations of quality of life and decrease performance status. Details of the study procedures were given on volunteers' information sheet. The benefits, confidentiality and voluntary participation features of the study were explained and written informed consent was obtained from all subjects. Ethical approval was obtained through the National Research Ethics Committee (Rwanda), and the University of the Witwatersrand-Johannesburg (South Africa), an institutional research partner to Kigali Health Institute.

### Procedures

Trained research associates administered a validated questionnaire, in which subjects reported changes in fat content affecting the face, posterior neck, breasts, abdomen, buttocks, upper and lower limbs [11]. The degree of body fat redistribution was rated as *absent *(score 0), *mild *(noticeable on close inspection, score 1), *moderate *(readily noticeable by the patient and the physician (score 2) or *severe *(readily noticeable to a casual observer, (score 3). The overall score was the mean of the scores given by the patients and a score assigned to each patient by a consensus of three clinicians working in the field of HIV/AIDS. Presence and rating of BFR was confirmed in all subjects by physical examination, in which 18% of HIV+ subjects (from self-reports) were excluded from HIV+ patients with moderate to severe BFR changes. For purposes of the current study, a clinical diagnosis was given to HIV patients with *moderate *(score 2) to *severe *(score 3) BFR, and an overall mean score of ≥ 18 on a validated 7-item body fat redistribution inventory for the face, neck, arms, breasts, abdomen, buttocks and legs, with 21 as a highest score. This was because self-perception of body changes in HIV patients with *mild *(score 1) changes may not have considerably influenced subjects' domains of quality of life. Subjects were divided into those with moderate-to-severe BFR (HIV+BFR, n = 50) and those with no BFR (HIV+noBFR, n = 50). Subjects' demographic characteristics including age, gender, occupation, education, data about HIV infection, type and duration of HAART and data on body fat changes were all obtained at the time of administration of the questionnaires.

Body composition and anthropometric measurements were measured by an experienced investigator. For consistency of data on skinfolds, one experienced investigator located and measured all skinfolds. Height (m), weight (Kg), body mass index (BMI) [calculated as weight (kg)/height (m)^2^], waist and hip circumferences (cm), waist-to-hip ratio (WHR) (calculated) were measured while the subject was standing, wearing light clothing and no shoes. Weight and height were measured to the nearest 0.1 kg and 0.1 cm respectively. Waist and hip circumferences were measured using an inelastic cloth tape. Waist circumference was measured at the narrowest circumference, halfway between the lowest ribs and iliac crests. Hip circumference was measured at the level of the anterior superior iliac spine, where this could be palpated, otherwise at the broadest circumference below the waist. Two measurements were taken, and when there was a more than 2 cm difference between the two, a third measurement was taken. The mean of the closest two measurements was used to calculate WHR. Mid-triceps, mid-biceps, supra-iliac and sub-scapular skinfolds were measured using Lange™ Skinfold Callipers. Measurements were read to the nearest 0.2 mm, and averaged for each skinfold site. The sum of skinfold measures were used to predict percentage body fat (% BFM) using derived equations and formulas [12,13], applicable on a black population [14].

### Outcome measures

Quality of life was measured using a summarized version of the World Health Organization's Quality of Life HIV instrument (WHOQOL-HIV) [15]. This is because although HIV infection is now a chronic illness and quality of life is an important outcome measure, mainly in HIV patients with body fat alterations, there are no validated instruments to assess the effects of body changes on quality of life in HAART-treated HIV population [1]. The instruments commonly used to assess QoL in HIV population include the Medical Outcome Study SF-36 form (MOS SF-36) widely used to assess QoL in general population with chronic diseases [16], the HIV/AIDS-Targeted Quality of Life instrument (HAT-QoL) [17] and the Multi-dimensional Quality of Questionnaire for HIV/AIDS (MQoL-HIV) [18]. However, most of these instruments were developed before the HAART era, and were tested in a single cultural setting usually in the developed world.

The WHOQOL-HIV instrument was developed for HIV population and has been shown to be valid in multi-cultural settings of heterogeneous social-economic strata including African countries [14]. Quality of life was assessed using the World Health Organization's Quality of Life HIV short form instrument (WHOQOL HIV BREF). The instrument contained items asking about how satisfied, how much, how completely, how bothered a person feels about different aspects of their life in the previous four weeks. The items were rated on a 5-point Likert interval scale where 1 indicated low negative perceptions and feelings, and 5 indicated very high positive perceptions and feelings. For example an item on HIV disclosure asked, "In the last four weeks, how much confident have you been about people knowing that you are HIV positive?" The available responses were: 1 (not at all), 2 (a little), 3 (a moderate amount), 4 (very much) and 5 (an extreme amount). However, since the QoL of HIV patients during the HAART era may fluctuate due to the positive effects of HAART, counterbalanced by effects of body fat changes [7], we included HIV HAART-specific domain of five facets specific for HIV patients during the HAART era. These items were generated from preliminary participants' interviews, clinicians, social scientists working in the field of HIV/AIDS and literature search [8,19]. These facets included feeling ashamed in public, problems of dressing style and size, feeling less confident about health, afraid of HIV disclosure and being embarrassed due to the impact of body fat alterations. Furthermore, to assess gender differences in the impact of BFR on QOL, subjects with BFR were asked to indicate how different body sites were affected by the presence of body fat redistribution. Subjects answered various questions such as 'in the past four weeks, how are the changes on your face affected by feeling ashamed in public places? Each item for different body sites had responses ranging from 1 (not at all), 2 (a little), 3 (a moderate amount), 4 (very much) and 5 (an extreme amount). Other domains include overall rating of quality of life, satisfaction with general health, and looking forward with hope to a better future. Therefore, the final instrument used in the current study was a short form of the World Health Organisation HIV instrument (WHOQOL HIV BREF), plus one domain of five facets to form 28 items.

### Statistical analysis

The data were analyzed using Fisher's Exact Test to determine the differences between groups, such as subject characteristics and body fat changes associated with quality of life. Continuous variables were analysed by paired t-tests. Nonparametric responses to the QoL questionnaire were analyzed using the Mann-Whitney Rank Sum Test (Kruskal-Wallis statistic). Scores were calculated and summarized so that facet scores were the mean of items in each facet. Domain scores were obtained by adding the facet mean scores in the respective domain, and dividing the number of facets in that domain, and multiplying by 4, so that the score range were from 4 to 20. Lower scores indicated poor self-perceived quality of life for the assessed health measure. Mean domain scores for each of the body parts affected were summarized by gender. Multiple linear regressions were performed to analyse the relationship between quality of life domain scores as dependent variable and independent variables between subject groups. All *p *values were 2-tailed, and statistical significance was set at 0.05. All data analysis was performed with STATA 8.1 statistical package (STATA Corp), and presented as mean ± SD, number (%) or median (interquartile range).

## Results

The demographic, body composition and clinical characteristics of the sample are presented in Table 1. Subject groups did not differ considerably with regard to age, gender, occupation, education and body mass index (BMI). HIV+BFR subjects received HAART for a significantly longer duration, and had larger waist but smaller hip circumferences and larger WHR than HIV+noBFR subjects. The sum skinfold and body fat mass did not differ between groups as subjects four-site skinfold thicknesses did not differ considerably (Table 1). Subjects were primarily receiving World Health Organisation (WHO)-recommended first line HAART regimens, with over 80% received stavudine, lamivudine and nevirapine, a widely used therapy in resource-limited regions of the world. The use of HAART regimen did not significantly differ between subject groups. CD4 cell counts for HIV+BFR men (333 ± 124) did not significantly differ from those of HIV+BFR women (366 ± 130 cells/μl) (p = 0.631). Also the CD4 cell counts for HIV+noBFR men (366 ± 133 cells/μl) were not different from those of HIV+BFR women (287 ± 118 cells/μl) (p = 0.413). HAART duration for HIV+BFR men was not different from that of HIV+BFR women [69.5 (30.8) versus 71.2 (26.9) months; p = 0.713]. Similarly HAART duration for HIV+noBFR men and women did not differ between groups [45.1 (13.3) versus 48.5 (15.5) months; p = 0.697].

**Table 1:** Sample characteristics, disease and body composition profiles

	**HIV+noBFR**	**HIV+BFR**	***p***
			
Age (years)	37.6 ± 6.3	37.5 ± 6.9	0.909
Number (females)	50 (60%)	50 (60%)	-
Occupation			
Public or private employment	6 (12%)	7 (14%)	0.685
Farming or livestock	29 (58%)	27 (54%)	0.896
Self-employed	9 (18%)	10 (20%)	0.985
Unemployed	6 (12%)	6 (12%)	1.000
Education			
≤ Secondary (High) school	20 (40%)	21 (42%)	0.986
Secondary (High) school	30 (60%)	29 (58%)	0.983
Tertiary (College) education	1 (2%)	-	-
Marital status			
Married	21 (42%)	9 (18%)	0.003
Separated	5 (10%)	6 (12%)	0.863
Widow or widower	18 (36%)	24 (48%)	0.060
Cohabiting	6 (12%)	11 (22%)	0.019
Smoking			
Yes	10 (20%)	7 (14%)	0.212
No	40 (80%)	43 (86%)	0.531
No. of years known to be HIV+			
≤ 4 years	18 (36%)	9 (18%)	0.007
5–10 years	22 (44%)	25 (50%)	0.231
≥ 11 years	10 (20%)	16 (32%)	0.012
CD4 cell count (cells/μl)	314 ± 160	347 ± 161	0.303
HAART duration (weeks)	45.7 (18.1)	62.7 (27.6)	<.0001
Body composition			
Body mass index (kg/m^2^)	25.5 ± 2.8	24.4 ± 2.7	0.064
Waist (cm)	85.0 ± 7.0	92.3 ± 6.9	<.0001
Hip (cm)	100.3 ± 4.3	93.7 ± 6.8	<.0001
Waist-to-hip ratio	0.85 ± 0.1	0.98 ± 0.1	<.0001
Skinfold (mm)			
Total skinfolds	60.4 ± 9.8	62.7 ± 12.9	0.378
Body fat mass (%)	29.1 ± 5.0	29.3 ± 4.3	0.846

Data expressed as mean ± SD, number (%) median (interquartile range); HIV+noBFR, HIV+ subjects with no body fat redistribution; HIV+BFR, HIV+ subjects with body fat redistribution.

### Quality of life scores by facets

HIV+BFR subjects were less satisfied with self-esteem and satisfaction with social life (4.1 ± 1.4 versus 2.1 ± 0.3; p = 0.003), body image and appearance (4.3 ± 0.1 versus 1.5 ± 0.2; p < .001), negative feelings about their job and routine house hold chores (4.5 ± 1.1 versus 3.1 ± 0.2; p = 0.027). HIV+BFR also reported more emotional stress than their HIV+noBFR counterparts (4.3 ± 1.4 versus 2.3 ± 0.7; p < .001). Subjects with body fat alterations further reported less satisfaction with interpersonal relationships (4.3 ± 1.2 versus 1.3 ± 1.2; p < .001), practical social support (4.3 ± 1.1 versus 1.1 ± 0.3; p < .001) and felt less respected and accepted by others (4.7 ± 1.1 versus 2.1± 1.5; p < .001). The wellbeing associated with spirituality, religion and beliefs (4.2 ± 1.2 versus 4.3 ± 1.0; p = 0.126) and satisfaction with sexual life (4.5 ± 1.7 versus 4.5 ± 0.2; p = 0.981) did not differ considerably between groups. However, HIV+BFR subjects reported more significant feeling ashamed in public places (4.5 ± 1.2 versus 1.1 ± 1.1), problems of dressing style and size (4.7 ± 1.6 versus 1.1 ± 0.7), less confident about their health (4.6 ± 1.4 versus 1.5 ± 1.2), fear of HIV disclosure (4.1 ± 1.8 versus 1.1 ± 1.3) and humiliation due to the impact of body fat alterations (4.1 ± 1.1 versus 1.1 ± 0.9) (p < .001).

### Body changes and quality of life

Table 2 lists subjects' mean scores on various domains of quality of life and effects of body fat alterations on QoL between African men and women. HIV+BFR subjects reported significantly less satisfaction with psychological wellbeing and social relationships. There were no considerable differences with regard to physical and independence domains of quality of life. Also, satisfaction with overall quality of life between subjects groups did not differ considerably (Table 2). Women with BFR reported less satisfaction with psychological wellbeing and social relationships. Women with BFR also reported more feeling ashamed in public and having problems of dressing style and size, fear of HIV disclosure and reported more embarrassment due to the impact of body changes (Table 2). There were no gender differences in physical functioning, independence wellbeing and overall rating of quality of life between subject groups. Marital status was associated with psychological (r^2 ^= 0.59; p < 0.05) and social relationships domains of QoL (r^2 ^= 0.49; p < 0.05), whereas age was associated with independence domain of QoL (r^2 ^= 0.69; p < 0.05). Gender was associated with psychological domain (r^2 ^= 0.68; p < 0.05) as well as HIV HAART-specific wellbeing (r^2 ^= 0.76; p < 0.05). On the other hand CD4 cell count (r^2 ^= 0.65; p < 0.05) and treatment duration (r^2 ^= 0.45; p < 0.05) were associated with HIV HAART-specific domain of wellbeing in HIV+ Rwandan subjects with body fat redistribution.

**Table 2:** Mean domain scores between subject groups, and by gender

‡ **Domains**	**HIV+noBFR**	**HIV+BFR**	***p***	**HIV+noBFR (men)**	**HIV+noBFR (women)**	***p***	**HIV+BFR (men)**	**HIV+BFR (women)**	***p***
						
Number	50	50		20	30		20	30	
Physical	18.3 (6.4)	17.2 (3.1)	0.352	17.9 (6.5)	18.6 (6.3)	0.876	17.4 (4.1)	16.9 (2.0)	0.653
Psychological	17.1 (5.8)	11.0 (2.4)	<.001	16.9 (5.6)	17.3 (5.9)	0.674	13.7 (1.9)	8.3 (2.9)	<.001
Independence	17.3 (6.7)	17.6 (5.1)	0.879	17.6 (6.6)	16.9 (6.8)	0.569	17.3 (6.1)	17.9 (4.1)	0.893
Social relationships	17.8 (5.1)	9.0 (3.2)	<.0001	17.9 (5.2)	17.6 (5.0)	0.898	11.1 (4.1)	6.9 (2.3)	<.001
*HIV HAART-specific	17.6 (5.7)	4.7 (4.2)	<.0001	17.9 (5.9)	17.3 (5.4)	0.672	6.3 (3.6)	3.1 (4.8)	<.001
† Overall aquality of life	15.7 (5.3)	15.5 (4.8)	0.889	15.6 (4.9)	15.8 (5.6)	0.935	15.8 (4.7)	15.2 (4.9)	0.598

Data expressed as median (interquartile range); ‡ Domain scores range from 4 to 20 and equals to the sum of facets divided by the number in each domain multiplied by 4; *Specific domain of quality of life for HAART-treated HIV patients; † other domains include overall rating of quality of life, satisfaction with general health, and looking forward with hope to a better future.

### Body changes, quality of life and gender

**Figure 1:**
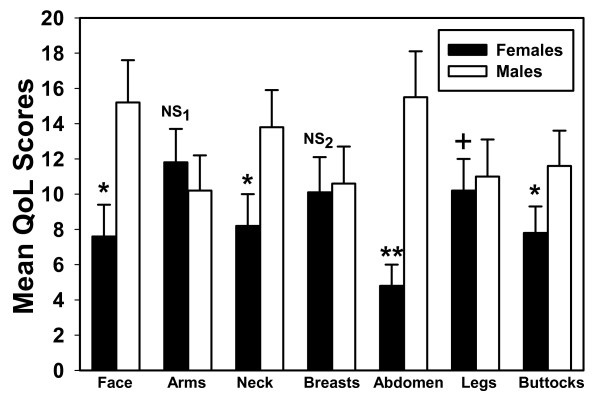
Quality of life and body changes by gender (n = 100; 60% females); Data expressed as median (interquartile range); on a summary scale of quality of life domain scores ranging from 4–20; *p < 0.05, **p < 0.001 versus male group; +p = 0.051, NS_1 _p = 0.745; NS_2 _p = 0.827.

Figure 1 presents the relationships between body fat alterations and summary scores of domains of quality of life by gender. Certain body changes significantly influenced HIV+ African women's mean quality of life scores than men. In particular, body fat alterations resulting in facial atrophy, lipohypertrophy of the neck (buffalo hump) and atrophy of the buttocks were significantly associated with higher mean quality of life scores. Women with BFR also reported less satisfaction with enlargement of the abdomen than men. There were no considerable differences between African men and women's perception of the impact of body fat alterations resulting in atrophy of the arms and enlargement of the breasts. There was a borderline difference in satisfaction, with women reporting less satisfaction with body changes resulting in atrophy of the legs than men (Figure 1).

## Discussion

Our findings indicate that HAART-treated HIV+ African men and women with BFR in Rwanda experience lower quality of life than their HIV-infected counterparts with no body fat alterations. Specifically, HIV+ subjects with body fat changes experienced greater psychological and social impairment of quality of life than those without body changes. Moreover, HIV+ patients with BFR experienced more social isolation and stigma due to feeling ashamed in public, embarrassment due to their body image and fear of forced HIV disclosure. To our knowledge, this is the first report on the relationship between body fat redistribution and quality of life in HAART-treated HIV+ African patients receiving WHO-recommended HAART. It is also the first report on the significant impact of HIV- and HAART-associated body changes on quality of life in HAART-treated HIV patients in sub-Saharan Africa. We believe these findings are of particular importance due to the consequences impaired quality of life may have on HAART adherence and treatment initiatives in sub-Saharan countries.

HIV-infected patients with BFR experienced social isolation and lack of self esteem due to poor body image and appearance. As the diagnosis with a chronic disease can have unfavourable impact on self esteem and interpersonal confidence [20], all HAART-treated HIV+ subjects regardless of BFR, experienced low quality of life on most domains of wellbeing. Although HIV+ patients with body changes had more favourable immunologic outcomes of higher CD4 counts, and were on treatment for a significantly longer duration, they experienced more psycho-social impairment of quality of life. Current CD4 counts or specific HAART regimen did not predict psychological and social wellbeing using multiple linear regression analysis, but predicted HIV HAART-specific domains of wellbeing associated with stigma and marginalisation due to HIV forced disclosure, symptoms that may impair psychological wellbeing. Other reports have shown that quality of life in HAART-treated HIV patients deteriorate due to the effects of body changes [21]. Psychological symptoms due to body fat changes may influence other domains of quality of life such physical and independence wellbeing [20]. However, we did not observe any difference in physical and independence wellbeing between HIV+ patients with or without body fat changes. Essentially, we cannot assume causality of the impact of body changes on quality of life over time due to the cross-sectional design of our study. Secondly, time since HIV diagnosis and severity of body fat changes did not predict any of the domains of quality of life using multiple linear regression analysis. However, our findings are in agreement with a report from Western countries, that physical domain of quality of life remains stable over time, whereas emotional stress and psychological domain deteriorate in HAART-treated HIV+ patients [22].

HIV+ patients with body changes felt more dishonoured due poor body image, particularly women who experienced greater marginalization as a result of changes on their face, neck, abdomen and buttocks. Women in the current study were more socially affected by having larger abdomen, which in Rwanda is associated with pregnancy, desired for by most African women but often discouraged due to the consequences of HIV infection. Furthermore, our findings indicate that changes in the face and reduction in size of the legs and buttocks were more associated with impairment in body image and appearance in women than men. However, both women and men were equally affected by reduction in the size of the arms and enlargement of breasts. Contrary to Western societies where women prefer a thin body size [23], African women, particularly those living with HIV, do not desire a slender body image. This is due to the cultural perception in most African countries, where feminine beauty is associated with larger body size, and the disclosure of HIV serostatus is associated with a slim body size. However, perception of body fat changes in the arms and breasts, may have been partly compensated for by the nature of dressing by most African women who often cover all their arms and chest. Thus, the quality of life for women was less affected by having thin arms and larger breasts. Women may have possibly been more affected by having both thin legs and buttocks due to less frequent dressing covering fully the legs especially on festival occasions and cultural perception of beauty for women with larger 'butts' and legs.

Although reports from Western countries demonstrate a relationship between morphologic changes and use of HAART with sexual dysfunction in HAART-treated HIV patients [24,25], we did not observe any difference in sexual satisfaction between HIV+ Rwandan patients with, and without body fat changes. Moreover, our subjects' perception of sexual satisfaction regardless of body fat changes was relatively high, and further studies including HIV seronegative subjects may ascertain these differences. However, in the current study multiple linear regression analysis indicated that both the duration of treatment and CD4 count were predictors of HIV and HAART-specific domain of quality of life. Subsequently, clinical and treatment of HIV such as longer use of HAART and higher CD4 counts, which predicts development of body fat alterations, appears also to be associated with quality of life in HIV+ Rwandan subjects with body fat changes.

Studies have demonstrated evidence for adverse effects of morphologic changes on body image and impairment of psychological and social relationships in HIV patients receiving HAART [26]. Our findings further demonstrate that HIV+ African men and women with BFR experience poor quality of life, due to forced disclosure of HIV+ diagnosis, stigmatization and isolation. This is consistent with other studies in which the quality of life of HIV+ patients with body changes if often more affected by fear of being recognised as HIV positive, resulting in impaired psychological wellbeing than effects on overall quality of life [27,28]. Therefore, body fat changes in HAART-treated HIV+ patients may result in negative changes in self esteem, interpersonal relationships, and raise questions regarding the overall benefits of HIV treatment in the African community. Others have shown that although HAART was keeping HIV+ patients alive, there was often tension between the desire for life sustaining treatment and optimal quality of life free from failure to conceal HIV serostatus and normal social interactions [6]. HAART- and HIV-associated morphologic changes are as stigmatizing as the wasting and skin lesions in the earlier years of the disease [8], and some of the affected individuals have described these changes as the 'Kaposi's sarcoma' of the 21^st ^century [29]. The severity of body changes affecting HAART-treated HIV+ Africans may compound the existing stigma and discrimination in HIV patients [30]. Initiatives to mitigate the effects of morphologic changes such as healthcare providers' psychological support and attention to patients' concerns regarding the effects of body fat changes, may potentially be beneficial, as BFR in HAART-treated patients affect medication adherence [10,31].

As initiatives to improve wider distribution of HAART in sub-Saharan Africa progress [3,4], the need to address the associated problems of stigma becomes increasingly important. Although the introduction of HAART has improved the quality of life of HIV+ patients by reducing HIV-related co-morbidities, the psychological and social consequences of chronic infection with HIV and body fat redistribution may lessen this positive impact. Therefore, treatment initiatives need to further enforce monitoring of the effects of HIV and HAART resulting in BFR in Africa, as the net benefit on the overall quality of life is considered to be a balance between decreased morbidity rates and the psycho-social symptoms of anxiety, depression and impaired self esteem [7].

Our study is limited by being a cross-sectional evaluation, which limits causal assumptions about the ways how body fat changes might affect psycho-social and independent wellbeing of HAART-treated HIV+ African patients. As quality of life may change over time, a longitudinal study could demonstrate better the relationships between effects of the disease progression and impact of BFR. Secondly, all subjects were on WHO-recommended HAART, provided freely by the government and bilateral organisations, and likely represented the general population, whose wellbeing is often affected by an array of financial constraints resulting in poverty which affect quality of life.

In summary, although the benefits of antiretroviral therapy cannot be underestimated, the psychological and social impact of the associated body fat changes cannot be ignored. Equally important to HIV treatment initiatives is to prioritize effective monitoring methods of HIV- and HAART-associated psychological and social consequences to maintain high levels of adherence. Effective and appropriate treatment programs need to adapt a more protracted approach, which embraces evaluation of self-perceived body changes and their determinants to improve provided care. Patient-centred care approach in which African patients are included in therapeutic decisions and paying attention to patients' perceptions of the effects of HAART, may contribute towards greater adherence to proposed interventions and develop a more stable quality of life continuum over time. An assessment of quality of life is integral to efficient treatment outcomes to evaluate long-term strategies that optimize the durability of response to antiretroviral therapy in sub Saharan countries.

## Competing interests

The author(s) declare that they have no competing interests.

## Authors' contributions

EM: design, enrolled participants, secured funding, drafted the manuscript, and oversaw the study. NJC: design, oversaw the study, reviewed and drafted manuscript. AS: design, oversaw the study, reviewed and drafted manuscript. All authors had equal responsibility for the decision to submit for publication.
